# BMI and Lifetime Changes in BMI and Cancer Mortality Risk

**DOI:** 10.1371/journal.pone.0125261

**Published:** 2015-04-16

**Authors:** Niloofar Taghizadeh, H. Marike Boezen, Jan P. Schouten, Carolien P. Schröder, E. G. Elisabeth de Vries, Judith M. Vonk

**Affiliations:** 1 University of Groningen, University Medical Centre Groningen, Department of Epidemiology, Groningen, the Netherlands; 2 University of Groningen, University Medical Centre Groningen, GRIAC research institute, Groningen, the Netherlands; 3 University of Groningen, University Medical Centre Groningen, Department of Medical Oncology, Groningen, the Netherlands; Hunter College, UNITED STATES

## Abstract

Body Mass Index (BMI) is known to be associated with cancer mortality, but little is known about the link between lifetime changes in BMI and cancer mortality in both males and females. We studied the association of BMI measurements (at baseline, highest and lowest BMI during the study-period) and lifetime changes in BMI (calculated over different time periods (i.e. short time period: annual change in BMI between successive surveys, long time period: annual change in BMI over the entire study period) with mortality from any cancer, and lung, colorectal, prostate and breast cancer in a large cohort study (n=8,645. Vlagtwedde-Vlaardingen, 1965-1990) with a follow-up on mortality status on December 31^st^ 2008. We used multivariate Cox regression models with adjustments for age, smoking, sex, and place of residence. Being overweight at baseline was associated with a higher risk of prostate cancer mortality (hazard ratio (HR) =2.22; 95% CI 1.19-4.17). Obesity at baseline was associated with a higher risk of any cancer mortality [all subjects (1.23 (1.01-1.50)), and females (1.40 (1.07-1.84))]. Chronically obese females (females who were obese during the entire study-period) had a higher risk of mortality from any cancer (2.16 (1.47-3.18), lung (3.22 (1.06-9.76)), colorectal (4.32 (1.53-12.20)), and breast cancer (2.52 (1.15-5.54)). We found no significant association between long-term annual change in BMI and cancer mortality risk. Both short-term annual increase and decrease in BMI were associated with a lower mortality risk from any cancer [all subjects: (0.67 (0.47-0.94)) and (0.73 (0.55-0.97)), respectively]. In conclusion, a higher BMI is associated with a higher cancer mortality risk. This study is the first to show that short-term annual changes in BMI were associated with lower mortality from any type of cancer.

## Introduction

Overweight and obesity are major public health problems in both developed and developing countries [1]. In the Netherlands, overweight and obesity rates have reached epidemic proportions, although they are still lower than in countries like the United States and UK. Obesity among Dutch adults increased from about 5% in the early 1980s to nearly 12% in 2011, while 41% was classified as overweight [2].

Several studies investigating the association between BMI and cancer suggest that a higher BMI can increase cancer incidence [3–5]. For example, in the United States, the proportion of all cancer deaths that can be attributed to overweight or obesity in adults older than 50 years was found to be 14% in males and 20% in females [6]. Moreover, maintaining a healthy weight has been proposed in cancer prevention strategies [3], suggesting a possible role of changes in BMI as well as baseline BMI regarding cancer risk.

The mechanisms underlying a possible link between BMI and cancer are still poorly understood [4]. However, insulin-like growth factor (IGF-1), sex-related hormones (e.g. oestrogen, progesterone, and androgens), and adipokines are factors that might explain this association [4,7]. Besides this poorly understood biological relationship between BMI levels and cancer, the effect of lifetime changes in BMI on cancer mortality risk has scarcely been studied.

The majority of previous studies were based on BMI measurements at only one time-point [8]. Moreover, the effect of BMI changes has been investigated mainly in relation to cancer incidence or all-cause mortality [9,10].

Another unanswered question is whether the associations between BMI and lifetime changes in BMI and cancer mortality risk differ between males and females, and evidence shows inconsistent results. For instance, one study showed a significant association between BMI and an increased risk of colorectal cancer in both males and females [11], while another study showed an increased risk only in males and not in females [12]. Although differences between sexes were suggested to be due to differences in fat distribution and a different hormonal system in males and females [6], the exact role of sex in the association between BMI and lifetime changes in BMI and cancer mortality risk is unknown.

Therefore we examined the association between BMI measurements (at baseline, highest and lowest during the study-period) and lifetime changes in BMI (calculated over different time periods (i.e. short time period: annual change in BMI between successive surveys, long time period: annual change in BMI over the entire study period) and cancer mortality risk in a large population-based cohort study. Additionally, we assessed whether the association between BMI and changes in BMI and cancer mortality risk is different for males and females. In our study, we investigated mortality from any type of cancer and from four common types of cancer (lung cancer, colorectal cancer, prostate cancer and breast cancer) in two Dutch communities (Vlagtwedde-Vlaardingen) [13–14]. The Vlagtwedde-Vlaardingen cohort, with a follow-up of over 40 years, offers a unique possibility to investigate the association between BMI and lifetime changes in BMI and subsequent cancer mortality, adjusting for potential confounders.

## Methods

### Ethics statement

The Committee on Human Subjects in Research of the University Medical Center Groningen and University of Groningen reviewed this study and affirmed the safety of the protocol and study design and all participants gave their written informed consent.

### Study population

We examined the association between BMI and mortality from cancer using the Vlagtwedde-Vlaardingen cohort study. The Vlagtwedde-Vlaardingen cohort study has been described in detail previously [13,14]. In brief, the study was set up as a general population-based cohort study on the epidemiology of pulmonary diseases in exclusively Caucasian individuals of Dutch descent. The study started in 1965, and participants had medical examinations every three years until the final survey in 1989/1990. In Vlaardingen, only participants who were included at baseline (1965 or 1969) were approached for follow-up, whereas in Vlagtwedde new subjects aged between 20 and 65 years were invited to participate at every survey. The number of surveys per subject ranged from one to eight (median number of surveys per subject: two). The final surveys were held in 1989 in Vlagtwedde and in 1990 in Vlaardingen. We updated the vital status of all participants in the Vlagtwedde-Vlaardingen study on December 31^st^, 2008, and evaluated mortality outcomes as follows: any cancer mortality and the four common types of cancer mortality (lung cancer, colorectal cancer, prostate cancer and breast cancer mortality), either as primary or secondary cause of death. Analyses on cause-specific mortality were performed at Statistics Netherlands (The Hague, the Netherlands).

### Medical characteristics

We collected data on age, sex, smoking habits, and place of residence using the Dutch version of the British Medical Research Council questionnaire [13].

### Body mass index (BMI)

We included only BMI measurements taken after the age of 20, thus assuming that subjects had reached their adult height by this age. The subject-specific mean heights of all surveys after age 20 were calculated. BMI was calculated as weight in kilograms divided by the square of the mean height in metres (kg/m^2^).

### Baseline BMI, highest BMI and lowest BMI during the study-period

In the analyses of BMI level, we used three BMI measurements: the BMI at baseline (i.e. the BMI at first available survey), the highest BMI during the study-period, and the lowest BMI during the study-period (S1 Fig). We placed the BMI measurements into three categories: 1) BMI < 25 kg/m^2^ (normal+ underweight, as reference); 2) BMI 25–30 kg/m^2^ (overweight), and 3) BMI > 30 kg/m^2^ (obese). Lowest and highest BMI during the study period were only determined in the subjects with at least two BMI measurements.

The subjects with a lowest BMI during the study-period higher than 30 kg/m^2^ were considered as chronic obese subjects. The subjects with a highest BMI during the study-period higher than 30 kg/m^2^ were considered as subjects who were obese at least once during the study-period.

### Lifetime changes in BMI

#### Long term annual change in BMI

We calculated the long-term annual change in BMI over the entire study period as the difference between BMI at last survey and BMI at baseline divided by the time interval (year of the last survey minus year of the baseline) (S1 Fig). Long-term annual change in BMI could only be calculated if subjects participated in at least two surveys. We were interested in comparing those subjects who had a decrease in their BMI, those who kept the same BMI and those who had a moderate and high increase in BMI. Therefore, we used four categories of long-term annual change in BMI: 1) Decrease < -0.02 kg/m^2^/year; 2) No change (reference) -0.02–0.02 kg/m^2^/year 3) Moderate increase 0.02–0.4 kg/m^2^/year, and 4) High increase > 0.4 kg/m^2^/year.

#### Short-term annual change in BMI

To define short-term annual change in BMI we calculated the annual changes in BMI between two successive surveys (S1 Fig). So, if a subject had performed 8 surveys, we calculated 7 annual changes in BMI. For each subject, the highest short-term BMI increase was defined as the highest annual increase in BMI between two successive surveys and the highest short-term BMI decrease was defined as the highest annual decrease in BMI between two successive surveys. Thus, for each subject only one short-term increase or decrease in BMI was calculated. Short-term annual changes in BMI were calculated only if subjects participated in at least three surveys. We included only subjects with at least three surveys, since if we included subjects with only two surveys, then the highest short-term increase would be the same as the highest short-term decrease. Moreover, the long-term change in BMI would then be equal to the short-term change for the subjects with only two surveys. The three surveys were successive but if a subject missed a survey in between the short-term change was calculated over a longer time period. We eliminated possible problems of categorization based on percentiles [15] by categorizing the subjects into no increase/decrease in BMI, a moderate increase/decrease in BMI and a high increase/decrease in BMI based on pre-defined cut-points.

The short-term annual increase in BMI was divided into three categories: 1) No increase (reference) < 0.10 kg/m^2^/year; 2) Moderate increase 0.10–0.50 kg/m^2^/year; 3) High increase > 0.50 kg/m^2^/year. The short-term annual decrease in BMI was also divided into three categories: 1) No decrease (reference) > −0.10 kg/m^2^/year; 2) Moderate decrease −0.10 - −0.50 kg/m^2^/year; 3) High decrease < −0.50 kg/m^2^/year.

Among all 8,465 subjects, 7,187 (84.9%) subjects had data available on baseline BMI measurements (taken after the age of 20) and on all included covariates. For 4,663 (55.1%) subjects a full dataset on highest and lowest BMI and on long-term annual change in BMI could be obtained. A full dataset on short-term annual changes in BMI was obtained for 3,864 (45.6%) subjects (S1–S4 Tables).

### Cancer mortality

Cancer mortality was classified according to the International Classification of Diseases (ICD) coding system. This classification was as follows: Any cancer (ICD 7: 140–239 and 294; ICD 8: 140–239; ICD 9: 140–239 and 288; ICD10: C00-C97, D00-D48), lung cancer (cancer of trachea, bronchus and lung) (ICD 7: 162, 163; ICD 8: 162, 163; ICD 9: 162, 163, 165; ICD10: C33, C34, C38, C39), cancer of colon and rectum (further referred to as colorectal cancer) (ICD 7: 153, 154; ICD 8: 153, 154; ICD 9: 153, 154; ICD 10 C18-C21), prostate cancer (ICD 7: 177; ICD 8: 185; ICD 9: 185 and ICD 10: C61), breast cancer (ICD 7: 170; ICD 8: 174; ICD 9:174, 175 and ICD10: C50).

### Statistical analyses

First, descriptive analyses of the subject characteristics and the mortality statistics were performed. Independent sample t-test and Chi-square test were used to determine significant differences between groups for continuous and categorical variables, respectively. Second, hazard ratios (HRs) associated with BMI level and changes in BMI for mortality from any cancer and any of the four common types of cancer were estimated using multivariate Cox regression. The Cox regression model was adjusted for age and smoking habits at baseline, sex, and place of residence. By performing stratified analyses, we estimated the BMI-associated risks for mortality from any cancer, lung cancer, and colorectal cancer for males and females separately and we tested for interactions. Subjects who died due to other causes were censored at their age of death. In the Cox regression, censoring took place when the subjects were still alive, were lost to follow-up, or died of causes other than cancer under study. Subjects for which the cause of dead was unknown were excluded from the analyses. The time variable in the Cox regression analysis was follow-up time since baseline. Log-minus-log plots were used to check if the proportional hazards assumption holds. P values less than 0.05 were considered significant.

## Results

### Characteristics

Among all 8,465 subjects, 4,505 (53.2%) were alive, 1,194 (14.1%) died due to cancer, 2,473 (29.2%) died due to another reason than cancer, 158 subjects (1.9%) died due to external causes such as an accident, suicide or homicide (Table 1). In 13 (0.1%) subjects the cause of death could not be determined, and 122 (1.5%) subjects were lost to follow-up. Of those subjects who died due to cancer, 275 (23.0%) died due to lung cancer, 134 (11.2%) died due to colorectal cancer, 83 (7.0%) died due to prostate cancer, and 117 (9.9%) died due to breast cancer.

**Table 1:** Characteristics at baseline according to vital status on December 31st 2008 in the general population of Vlagtwedde-Vlaardingen, during 40 years of follow-up (n = 8645).

Characteristics	Alive (A)(n = 4505)	Died due to cancer (DC) (n = 1194)	Died but not due to cancer (DNC) (n = 2473)	Died due to external causes (n = 158)	Lost to follow-up (n = 122)	p-value DC vs. A	p-value DC vs.DNC
All subjects (%)	53.2	14.1	29.2	1.9	1.5		
Men (%)	48.8	58.3	54.8	63.9	57.4	<0.01	0.04
Age in years, mean (sd)	30.2 (10.2)	45.9 (11.1)	50.1 (9.6)	43.6 (13.7)	33.2 (13.3)	<0.01	<0.01
Smoking (%)							
Never smokers	38.0	33.2	39.8	35.9	38.8	<0.01	<0.01
Ever smokers	62.0	66.8	60.2	64.1	61.2		
BMI levels (%)							
Normal	60.9	39.4	32.4	50.8	64.7	<0.01	<0.01
Overweight	31.8	44.7	49.5	37.9	29.3		
Obese	7.3	15.9	18.0	11.3	6.0		
BMI at baseline, kg/m^2^	24.8 (3.5)	26.3 (3.8)	26.9 (4.0)	25.4 (3.6)	23.7 (3.4)	<0.01	<0.01
Highest BMI during the study-period	26.4 (3.9)	27.9 (4.0)	28.0 (4.2)	26.8 (4.2)	25.3 (3.5)	<0.01	0.90
Lowest BMI during the study-period	24.1 (3.2)	25.2 (3.6)	25.1 (3.6)	24.1 (3.3)	24.3 (3.2)	<0.01	0.84
Long term annual changes in BMI, kg/m2/yr	0.1 (0.2)	0.1 (0.2)	0.0 (0.3)	0.1 (0.3)	0.2 (0.4)	<0.01	0.01
Shor-term annual changes in BMI, kg/m2/yr							
Highest increase	0.5 (0.4)	0.4 (0.4)	0.4 (0.5)	0.3 (0.4)	0.4 (0.5)	<0.01	0.17
Highest decrease	-0.3 (0.5)	-0.3 (0.4)	-0.4 (0.5)	-0.3 (0.5)	-0.1 (0.6)	0.60	<0.01

All subjects: n = 8452, in 13 subjects the cause of death could not be determined. BMI levels: Normal = BMI <25 kg/m^2^, Overweight = BMI 25–30 kg/m^2^. Obese = BMI > 30 kg/m^2^. Long-term annual change in BMI: The difference between BMI at last survey and baseline divided by the time interval (year of the last survey minus year of the baseline). Short-term annual changes in BMI: Highest increase = Highest annual increase in BMI between two successive surveys, Highest decrease = Highest annual decrease in BMI between two successive surveys. P-value calculated by Chi- square or t-test. Data on BMI levels and changes in BMI are shown as mean (sd).

The characteristics of subjects who died due to specific types of cancer are shown in S5 Table. The mean age at baseline of the subjects who died due to cancer was 45.9 (standard deviation (sd) = 11.1) years, and 58% were males. Subjects who died due to cancer were more often ever smokers (66.8%) compared to subjects who were alive or died due to causes other than cancer. Subjects who died due to cancer had significantly higher BMI levels at baseline compared to subjects who were alive, but had lower BMI levels compared to subjects who died due to causes other than cancer (Table 1).

The median (range) time interval between the BMI measurements used to calculate the long-term annual BMI change was 16 years (3–25) and 3,303 subjects had observations more than 10 years apart.

### Baseline BMI, highest BMI and lowest BMI during the study-period

**Fig 1:**
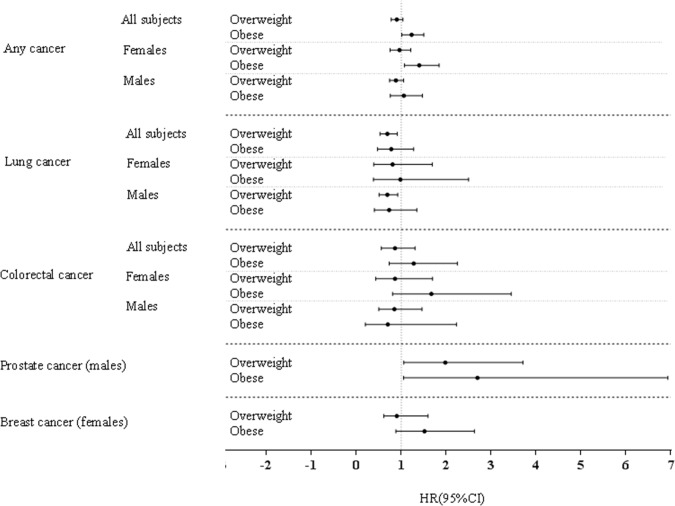
Hazard ratio of different BMI categories at baseline (Normal (reference category), overweight, and obese) for any and specific types of cancer mortality.

Associations between baseline BMI and cancer mortality are shown in Table 2. Being overweight was associated with a higher risk of mortality from prostate cancer [males: (2.22 (1.19–4.17), but a decreased risk of mortality from lung cancer [all subjects: (0.69 (0.53–0.91), and males: (0.69 (0.51–0.92)]. Obesity at baseline was associated with a higher risk of mortality from any type of cancer [all subjects: (HR) (95% CI) = 1.23 (1.01–1.50)), and females: (1.40 (1.07–1.84))] and prostate cancer [males (3.33 (1.31–8.46)] (Table 2, S1 and S6 Tables, Fig 1). No significant interactions were observed between baseline BMI and sex on risk of cancer mortality (S6 Table).

**Table 2:** Number of subjects and hazard ratio (with 95% confidence interval) of BMI at baseline for mortality from any cancer, lung cancer, colorectal cancer, among all 7187 subjects, prostate cancer, among 3718 males and breast cancer among 3469 females in Cox regression with adjustment for age, smoking habits, and place of residence.

BMI level at baseline	Any cancer		Lung cancer		Colorectal cancer		Prostate cancer		Breast cancer	
	Nevents/censored	HR (95% CI)	Nevents/censored	HR (95% CI)	Nevents/censored	HR (95% CI)	Nevents/censored	HR (95% CI)	Nevents/censored	HR (95% CI)
All subjects										
Normal	387/3082	1	114/3355	1	40/3429	1	13/1817	1	35/1604	1
Overweight	446/2431	0.90 (0.78–1.04)	99/2778	**0.69 (0.53–0.91)**	51/2826	0.86 (0.56–1.31)	41/1604	**2.22 (1.19–4.17)**	39/1193	0.90 (0.61–1.60)
Obese	158/683	**1.23 (1.01–1.50)**	19/822	0.78 (0.47–1.28)	22/ 819	1.28 (0.73–2.25)	7/236	**3.33 (1.31–8.46)**	29/569	1.52 (0.88–2.63)

Normal = BMI <25 kg/m^2^, overweight = BMI 25–30 kg/m^2^, obese = BMI > 30 kg/m^2^. Statistically significant results are shown in bold.

**Fig 2:**
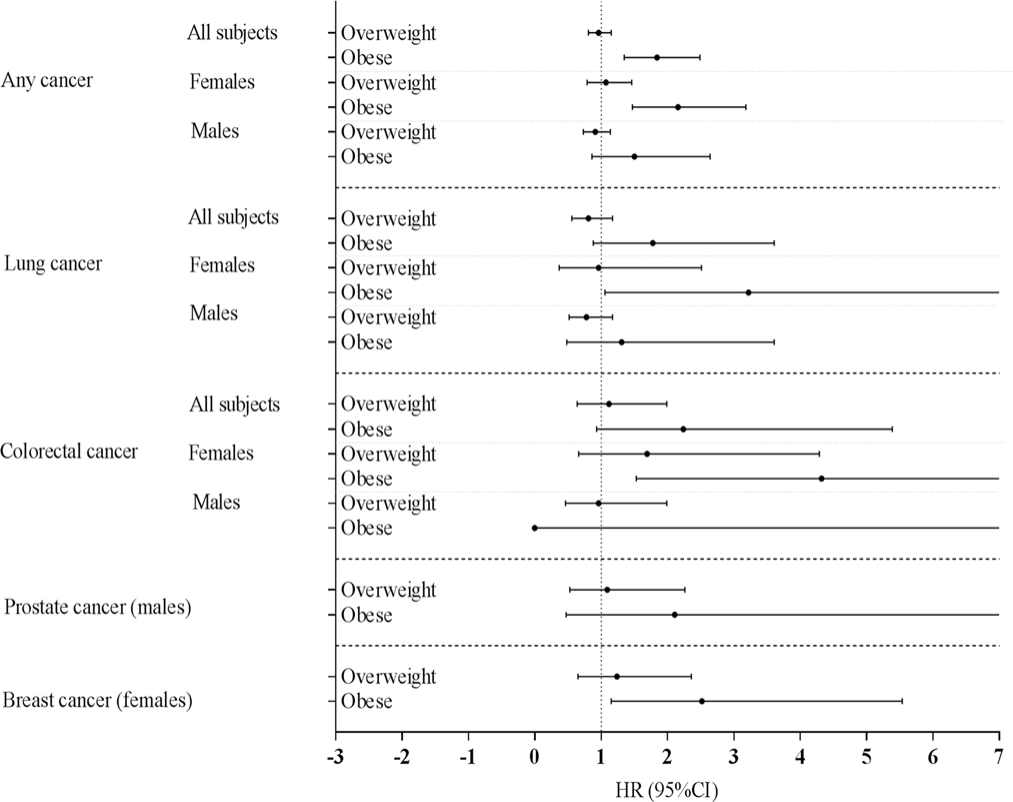
Hazard ratio of lowest BMI during follow-up for any and specific types of cancer mortality, relative to normal BMI.

Females who were overweight or obese at least once during the study-period were more likely to die from any type of cancer (overweight females: 1.59 (1.06–2.38)) and obese females: (1.87 (1.21–2.89)) (S2 and S7 Tables, upper panel). Being overweight at least once during the study-period was associated with a decreased risk of mortality from colorectal cancer among males (0.40 (0.18–0.93)) (S2 and S7 Tables). Being overweight at least once during the study-period significantly interacted with sex on risk of mortality due to any cancer ((HR) (95% CI) = 0.58 (0.36–0.93), p-value: 0.023 and colorectal cancer ((HR) (95% CI) = 0.09 (0.01–0.77), p-value: 0.028). This indicates that the association between being overweight at least once during the study-period and any cancer or colorectal cancer mortality is significantly different between males and females; the effect was more pronounced in females (S7 Table). Chronic obesity was associated with a higher risk of mortality from any type of cancer among all subjects (1.84 (1.35–2.49)) (Table 3, lower panel, Fig 2).

**Table 3:** Number of subjects and hazard ratio (with 95% confidence interval) of highest and lowest BMI during the study-period for mortality from any cancer, lung cancer, colorectal cancer among all 4663 subjects, prostate cancer among 2448 males and breast cancer among 2215 females in Cox regression with adjustment for age, smoking habits, and place of residence.

Highest BMI level	Any cancer		Lung cancer		Colorectal cancer		Prostate cancer		Breast cancer	
	Nevents/censored	HR (95% CI)	Nevents/censored	HR (95% CI)	Nevents/censored	HR (95% CI)	Nevents/censored	HR (95% CI)	Nevents/censored	HR (95% CI)
All subjects										
Normal	116 /1210	1	35 /1291	1	11 /1315	1	2 /645	1	11 /668	1
Overweight	308/2083	0.99 (0.79–1.23)	77/2314	0.77 (0.51–1.15)	27 /2364	0.79 (0.39–1.62)	24/1399	3.56 (0.84–15.14)	22/946	1.14 (0.53–2.43)
Obese	137/809	1.27 (0.99–1.65)	23 /923	0.80 (0.47–1.37)	18 /928	1.45 (0.66–3.18)	6/372	3.89 (0.77–19.60)	18/550	1.55 (0.69–3.49)
**Lowest BMI level**	**Any cancer**		**Lung cancer**		**Colorectal cancer**		**Prostate cancer**		**Breast cancer**	
All subjects										
Normal	298 /2633	1	80/2851	1	25 /2906	1	15 /1550	1	22 /1344	1
Overweight	210 /1276	0.96 (0.81–1.15)	46/1440	0.81 (0.56–1.17)	24 /1462	1.12 (0.64–1.99)	15/804	1.24 (0.60–2.56)	19 /648	1.24 (0.65–2.36)
Obese	53 /193	**1.84 (1.35–2.49)**	9 /237	1.78 (0.88–3.61)	7 /239	2.24 (0.93–5.39)	2 /62	3.18 (0.72–14.08)	10 /172	**2.52 (1.15–5.54)**

BMI levels: Normal = BMI <25 kg/m^2^, overweight = BMI 25–30 kg/m^2^, obese = BMI > 30 kg/m^2^. Statistically significant results are shown in bold.

Chronically obese females, obese during the entire follow up, had a higher risk of mortality from any cancer (2.16 (1.47–3.18)), from lung cancer (3.22 (1.06–9.76); p = 0.04), colorectal cancer (4.32 (1.53–12.20)), and breast cancer (2.52 (1.15–5.54)) (Table 3, S2 and S7 Tables lower panel, Fig 2). We found no significant association between chronic obesity and risk of mortality from any type of cancer among males. No significant interactions were observed between chronic obesity and sex on risk of cancer mortality (S7 Table). In addition, sensitivity analyses, excluding the underweight subjects or the ever smokers showed comparable results (results not shown).

### Lifetime changes in BMI

#### Long-term annual change in BMI

There were no significant associations between long-term annual change in BMI and cancer mortality risk (S3 and S8 Tables). No significant interactions were observed between long-term annual change in BMI and sex on risk of cancer mortality (S9 Table).

#### Short-term annual changes in BMI

**Fig 3:**
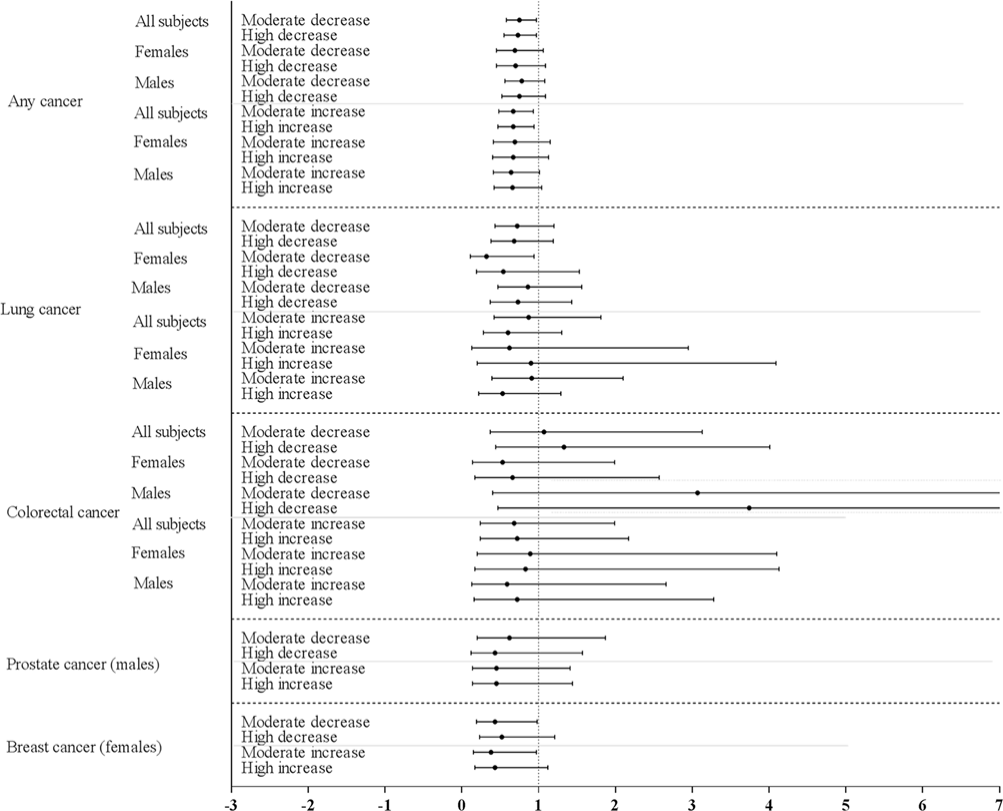
Hazard ratio of short-term annual changes in BMI (moderate to high increase or decrease) for any and specific types of cancer mortality, relative to no change in BMI.

We observed several significant associations between short-term annual changes in BMI and cancer mortality risk. A moderate short-term annual increase in BMI (an increase in BMI of 0.10–0.50 kg/m^2^/year) was associated with a decreased risk of mortality from any cancer (0.67 (0.48–0.93)) and breast cancer (0.38 (0.15–0.97)). A high short-term annual increase in BMI (an increase in BMI of > 0.50 kg/m^2^/year) was associated with a decreased risk of mortality from any cancer among all subjects (0.67 (0.47–0.94)) (Table 4, upper panel, S4 Table, Fig 3). A moderate short-term annual decrease in BMI was associated with a decreased risk of mortality from any cancer among all subjects (0.75 (0.58–0.97)). Additionally, a moderate short-term annual decrease in BMI was associated with a decreased risk of mortality from lung cancer (0.32 (0.11–0.94)), and breast cancer among females (0.43 (0.19–0.98)) (Table 4, S10 Table). A high short-term annual decrease in BMI was associated with a decreased risk of mortality from any cancer (0.73 (0.55–0.97)), (Table 4, lower panel, S4 Table, Fig 3). No significant interactions were observed between short-term annual changes in BMI and sex on risk of cancer mortality (S10 Table).

**Table 4:** Number of subjects and hazard ratio (with 95% confidence interval) of short-term annual changes in BMI (highest increase and highest decrease in BMI between two subsequent observations) for mortality from all cancer, lung cancer, colorectal cancer among all 3864 subjects.

Highest short-term annual increase in BMI, n (%)	Any cancer		Lung cancer		Colorectal cancer		Prostate cancer		Breast cancer	
	Nevents/censored	HR (95% CI)	Nevents/censored	HR (95% CI)	Nevents/censored	HR (95% CI)	Nevents/censored	HR (95% CI)	Nevents/censored	HR (95% CI)
All subjects										
No increase	40 /156	1	8 /188	1	4 /192	1	4 /87	1	6 /99	1
Moderate increase	237 /1605	**0.67 (0.48–0.93)**	68 /1774	0.87 (0.42–1.81)	23 /1819	0.68 (0.24–1.99)	14 /1017	0.33 (0.11–1.00)	16/795	**0.38 (0.15–0.97)**
High increase	198 /1628	**0.67 (0.47–0.94)**	40 /1786	0.60 (0.28–1.30)	18 /1808	0.72 (0.24–2.17)	11/917	0.36 (0.11–1.14)	18 /880	0.43 (0.17–1.12)
**Highest short-term annual decrease in BMI, n (%)**	**Any cancer**		**Lung cancer**		**Colorectal cancer**		**Prostate cancer**		**Breast cancer**	
	Nevents/censored	HR (95% CI)	Nevents/censored	HR (95% CI)	Nevents/censored	HR (95% CI)	Nevents/censored	HR (95% CI)	Nevents/censored	HR (95% CI)
All subjects										
No decrease	76/619	1	20/675	1	4 /691	1	4 /409	1	9 /273	1
Moderate decrease	256 /1722	**0.75 (0.58–0.97)**	64 /1914	0.72 (0.43–1.20)	24/1954	1.07 (0.37–3.13)	19/1093	0.84 (0.28–2.51)	16 /850	**0.43 (0.19–0.98)**
High decrease	143 /1048	**0.73 (0.55–0.97)**	32/1159	0.68 (0.38–1.19)	17/1174	1.33 (0.44–4.01)	6 /519	0.57 (0.16–2.06)	15/651	0.52 (0.23–1.21)

Prostate cancer among 2050 males and breast cancer among 1814 females in Cox regression with adjustment for age, smoking habits, and place of residence. Highest increase in BMI: No increase = < 0.10 kg/m^2^/yr, moderate increase = 0.10–0.50 kg/m^2^/yr, high increase = > 0.50 kg/m^2^/yr. Highest decrease in BMI: No decrease = > -0.10 kg/m^2^/yr, moderate decrease = -0.10- -0.50 kg/m^2^/yr, high decrease = < -0.50 kg/m^2^/yr. Statistically significant results are shown in bold.

Sensitivity analyses, excluding the ever smokers showed comparable results (results not shown). Furthermore, additional adjustment for baseline BMI did not change the results (results not shown).

## Discussion

This is the first large cohort study in a general population investigating the association of BMI levels and long-term and short-term annual changes in BMI with any cancer mortality and four common types of cancer mortality. We showed that higher BMI was associated with a higher mortality from any type of cancer. Chronic obesity was associated with higher mortality from any type of cancer among females. We found no significant association between annual change in BMI and cancer mortality. We observed that a moderate to high annual short-term increase in BMI as well as a moderate to high annual short-term decrease in BMI was associated with lower mortality from any type of cancer.

Obesity was associated with an increased risk of mortality from any cancer and prostate cancer in our study. Overweight was also associated with a decreased risk of prostate cancer mortality. So far, studies on the association between BMI and prostate cancer have shown modest positive associations with prostate cancer incidence [7,16] and stronger positive associations with prostate cancer mortality [17,18]. Potential biological mechanisms underlying the association between BMI and prostate cancer could act through insulin, IGF-I, and testosterone [17,19]. For example, obesity is associated with increased levels of insulin and free IGF-I, which changes the cellular environment favoring (prostate) cancer development [7,20]. Increased BMI is associated with decreased free testosterone levels in males [21]. Although a lower level of testosterone does not seem to be associated with an increased risk of prostate cancer [22], it does appear to be associated with more severe disease with a worse prognosis [23].

We found a lower risk of mortality from lung cancer among overweight subjects, especially males. Several other studies consistently found this inverse association and suggested that this association is independent of smoking and weight loss because of preclinical disease [24–27]. To test this suggestion we performed sensitivity analyses excluding the subjects who were ever smokers or died within 5 years of the baseline visit. These analyses gave similar results as the main analysis (results not shown) and thus confirmed that the observed associations are independent of smoking or weight loss because of preclinical disease. The biological mechanism explaining this inverse association is not yet clear [23,25]. Among smokers, a possible explanation for this inverse association between BMI and lung cancer is that leaner smokers have higher concentrations of blood cotinine [25] (a metabolite of nicotine indicating tobacco smoke exposure), which may induce tumour promotion [28].

Another interesting finding was that the association between chronic obesity and cancer mortality was only present in females and not in males. This sex difference may be related to differences in fat distribution between males and females. Males with increased BMI tend to have central adiposity, whereas females tend to have general overweight [26]. This may affect cancer risk differently. In addition, a high BMI is associated with higher levels of endogenous sex hormones, but there are some differences in these associations between males and females. For example, in severely obese males testosterone levels decrease due to a strong reduction in gonadotropic stimulation of testicular testosterone synthesis [4], whereas in obese females testosterone levels increase [4]. Further research into these sex-specific associations between BMI and sex hormones and cancer risk may shed more light on this.

Another explanation for the sex difference in the association between chronic obesity and cancer mortality may be that increased BMI is associated very strongly with mortality from other chronic diseases such as cardiovascular disease, especially in males [25]. It is possible that more males than females have already died from cardiovascular disease before they could develop cancer. This would decrease the HR for chronic obesity on cancer mortality only in males. A competing risk analysis [29] indeed showed that chronic obesity was associated with a higher risk for cancer mortality in females and with a higher risk for cardiovascular mortality in males (see S11 Table).

We found no significant association between long-term annual change in BMI and cancer mortality. However, the study power is compromised in our study, because of low number of subjects in which the long-term annual change in BMI could be obtained, especially when specific types of cancer mortality and gender were considered. Surprisingly, we observed that a moderate to high short-term annual increase in BMI and a moderate to high short-term annual decrease in BMI are both associated with a decreased risk of mortality from any type of cancer among all subjects, and from breast cancer among females. In line with this, Michels et al found that both weight loss and weight gain are associated with decreased breast cancer incidence among premenopausal women. However, these associations were not statistically significant [30]. Some other studies suggested that the association between BMI and an increased risk of breast cancer might be limited to pre-menopausal hormone receptor-negative breast cancer [31–32]. This suggests that the association between BMI and cancer varies by cancer subtype, which warrants future studies.

Since higher BMI is associated with increased cancer risk, the finding that short-term decreases are associated with a decreased cancer risk is not surprising. Tee et al. in a recent systematic review and meta-analysis reported that weight loss with surgical intervention, is associated with decreased risk of cancer only among females [33]. The fact that a sudden increase in weight is also associated with a decreased risk of cancer mortality might be explained by the fact that the cause of the specific weight gain might be a protective factor for cancer, not the weight gain itself.

In our study, for females, this short-term weight gain may be due to pregnancy, which is a protective factor for breast cancer [34]. In smokers, short-term weight gain may be due to quitting smoking [35], which also decreases the risk of cancer.

The strengths of our study include the large number of subjects (n = 8,645) sampled from the general population, with a long follow-up and high follow-up rate: 98.5% of the included subjects could be traced back [36]. This enabled us to investigate lifetime changes in BMI, as well as chronic obesity as risk factors for cancer mortality. Moreover, we did not use self-reported weight and height, but weight and height measured in a standardized way during all surveys, thereby eliminating measurement bias in the BMI data, and misclassification of subjects into incorrect BMI category, which occurs when you ask people for their weight instead of measuring it [37].

Another strength of the study is that we studied the association between BMI and cancer mortality in four common types of cancer (lung cancer, colorectal cancer, prostate cancer and breast cancer), while previous studies investigated only one type of cancer or rare cancers (e.g. gastric cancer and gallbladder cancer). Finally, our results include sex-specific estimates, which were assessed in two geographical regions (Vlagtwedde and Vlaardingen). Finally, BMI measurements and changes in BMI were determined prior to cancer diagnosis, thus in apparently healthy subjects.

The lack of information on alcohol consumption, and physical activity may be considered a limitation in our study. Evidence shows that alcohol consumption may modify the effect of other exposures including BMI on risk of cancer [38]. Physical activity may be causally related to BMI, and thus plays an important role in the etiology of different types of cancer. Moreover, it acts through multiple biological mechanisms that influence the cancer development [39]. Therefore, further studies considering different confounders are needed to clarify the association between BMI, and changes in BMI and risk of cancer mortality.

Clearly, our study is highly relevant and timely given the rapidly spreading global obesity epidemic. In addition, our results underscore the importance of strategies to prevent and reduce overweight and obesity.

## Supporting Information

S1 Fig BMI measurements in Vlagtwedde-Vlaardingen study. (TIF)

S1 Table Number of subjects and follow-up times (FU) of subjects included in the analyses on the associations between BMI at baseline and mortality due to any cancer, lung cancer, colorectal cancer, prostate cancer, and breast cancer, in a general population of Vlagtwedde-Vlaardingen during 40 years of follow-up. Normal = BMI <25 kg/m2, Overweight = BMI 25–30 kg/m2, Obese = BMI > 30 kg/m2.
(DOCX)

S2 Table Number of subjects and follow-up times (FU) of subjects included in the analyses on the associations between highest and lowest BMI level during study-period and mortality due to any cancer, lung cancer, colorectal cancer, prostate cancer, and breast cancer, in a general population of Vlagtwedde-Vlaardingen during 40 years of follow-up. Normal = BMI <25 kg/m2, Overweight = BMI 25–30 kg/m2, Obese = BMI > 30 kg/m2.
(DOC)

S3 Table Number of subjects and follow-up times (FU) of subjects included in the analyses on the associations between long-term annual change in BMI and mortality due to any cancer, lung cancer, colorectal cancer, prostate cancer, and breast cancer, in a general population of Vlagtwedde-Vlaardingen during 40 years of follow-up. Decrease = < .0.02 kg/m2/yr, No change = -0.02–0.02 kg/m2/yr, Moderate increase = 0.02–0.4 kg/m2/yr, High increase = > 0.4 kg/m2/yr.
(DOC)

S4 Table Number of subjects and follow-up times (FU) of subjects included in the analyses on the associations between short-term annual changes in BMI and mortality due to any cancer, lung cancer, colorectal cancer, prostate cancer, and breast cancer, in a general population of Vlagtwedde-Vlaardingen during 40 years of follow-up. Highest short-term annual increase in BMI: No increase = < 0.10 kg/m2/yr, Moderate increase = 0.10–0.50 kg/m2/yr, High increase = > 0.50 kg/m2/yr. Highest short-term annual decrease in BMI: No decrease = > -0.10 kg/m2/yr, Moderate decrease = -0.10- -0.50 kg/m2/yr, High decrease = < -0.50 kg/m2/yr.
(DOC)

S5 Table Characteristics at baseline, for subjects who died due to lung cancer, colorectal cancer, prostate cancer, and breast cancer in a general population of Vlagtwedde-Vlaardingen during 40 years of follow-up. Long-term annual change in BMI: The difference between BMI at last survey and baseline divided by the time interval (year of the last survey minus year of the baseline). Short-term annual changes in BMI: Highest increase = Highest annual increase in BMI between two successive surveys, Highest decrease = Highest annual decrease between two successive surveys. Data on BMI levels and changes in BMI are shown as mean (sd).
(DOC)

S6 Table Hazard ratio (with 95% confidence interval) of BMI at baseline for mortality from any cancer, lung cancer, colorectal cancer, among 3718 males and 3469 females in Cox regression with adjustment for age, smoking habits, and place of residence. Stratification according to sex and interactions are shown. Normal = BMI <25 kg/m2, overweight = BMI 25–30 kg/m2, obese = BMI > 30 kg/m2. Statistically significant results are shown in bold.
(DOC)

S7 Table Hazard ratio (with 95% confidence interval) of highest and lowest BMI during the study-period for mortality from any cancer, lung cancer, colorectal cancer among 2448 males and 2215 females in Cox regression with adjustment for age, smoking habits, and place of residence. Stratification according to sex and interactions are shown. BMI levels: Normal = BMI <25 kg/m2, overweight = BMI 25–30 kg/m2, obese = BMI > 30 kg/m2. Statistically significant results are shown in bold. NA: Not Available, no mortality in this category.
(DOC)

S8 Table Hazard ratio (with 95% confidence interval) of long-term annual change in BMI categories for mortality from all cancer, lung cancer, colorectal cancer among all 4663 subjects. Prostate cancer among 2448 males and breast cancer among 2215 females in Cox regression with adjustment for age, smoking habits, and place of residence. Long-term annual change in BMI: Decrease = < -0.02 kg/m2/yr, no change = -0.02–0.02 kg/m2/yr, moderate increase = 0.02–0.4 kg/m2/yr, high increase = > 0.4 kg/m2/yr. NA: Not Available, no mortality in this category.
(DOC)

S9 Table Hazard ratio (with 95% confidence interval) of long-term annual change in BMI over the entire study period categories for mortality from all cancer, lung cancer, colorectal cancer among 2448 males and 2215 females in Cox regression with adjustment for age, smoking habits, and place of residence. Stratification according to sex and interactions are shown. Long-term annual change in BMI: Decrease = < -0.02 kg/m2/yr, no change = -0.02–0.02 kg/m2/yr, moderate increase = 0.02–0.4 kg/m2/yr, high increase = > 0.4 kg/m2/yr. NA: Not Available, no mortality in this category.
(DOC)

S10 Table Hazard ratio (with 95% confidence interval) of short-term annual changes in BMI (highest increase and highest decrease in BMI between two subsequent observations) for mortality from all cancer, lung cancer, colorectal cancer among 2050 males 1814 females in Cox regression with adjustment for age, smoking habits, and place of residence. Stratification according to sexes and interactions are shown. Normal = BMI <25 kg/m2, Overweight = BMI 25–30 kg/m2, Obese = BMI > 30 kg/m2.
(DOC)

S11 Table Competing risk analysis on the association between the lowest BMI during follow-up and cancer, and cardiovascular mortality in males and females. Highest increase in BMI: No increase = < 0.10 kg/m2/yr, moderate increase = 0.10–0.50 kg/m2/yr, high increase = > 0.50 kg/m2/yr. Highest decrease in BMI: No decrease = > -0.10 kg/m2/yr, moderate decrease = -0.10- -0.50 kg/m2/yr, high decrease = < -0.50 kg/m2/yr. Statistically significant results are shown in bold.
(DOC)
