# Supplementary figures and images for: BMI and Lifetime Changes in BMI and Cancer Mortality Risk
Source: PLoS One. 2015 Apr 16;10(4):e0125261. doi: 10.1371/journal.pone.0125261 (PMC4399977; doi:10.1371/journal.pone.0125261)

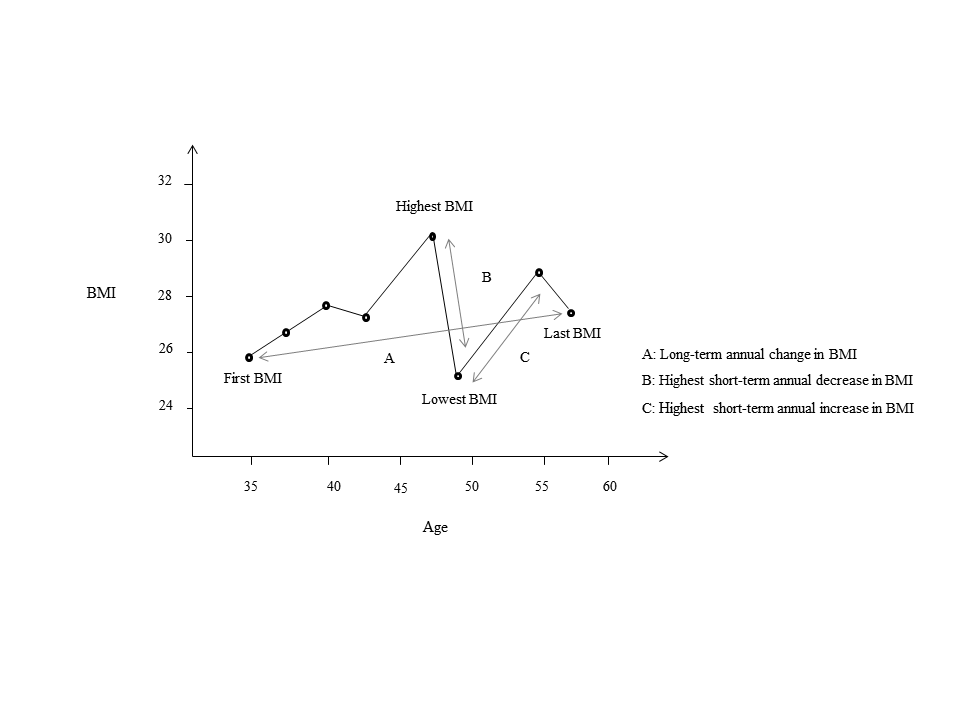

Supplement: S1 Fig — (TIF) [file pone.0125261.s001.tif]
