# Supplementary material for: BMI and Lifetime Changes in BMI and Cancer Mortality Risk
Source: PLoS One. 2015 Apr 16;10(4):e0125261. doi: 10.1371/journal.pone.0125261 (PMC4399977; doi:10.1371/journal.pone.0125261)
Supplement: S1 Table — Normal = BMI <25 kg/m2, Overweight = BMI 25–30 kg/m2, Obese = BMI > 30 kg/m2. (DOCX) [file pone.0125261.s002.docx]

**S1 Table- Number of subjects and follow-up times (FU) of subjects included in the analyses on the associations between BMI at baseline and mortality due to any cancer, lung cancer, colorectal cancer, prostate cancer, and breast cancer, in a general population of Vlagtwedde-Vlaardingen during 40 years of follow-up.**

| **BMI level at baseline** | **Any cancer** | | **Lung cancer** | | **Colorectal cancer** | | **Prostate cancer** | | **Breast cancer** | |
| --- | --- | --- | --- | --- | --- | --- | --- | --- | --- | --- |
|  |  |  |  |  |  |  |  |  |  |  |
|  | **median FU (yr)**  **events/censored** | **N (%) events/censored** | **median FU (yr) events/censored** | **N (%) events/censored** | **median FU (yr) events/censored** | **N (%) events/censored** | **median FU (yr) events/censored** | **N (%) events/censored** | **median FU (yr) events/censored** | **N (%) events/censored** |
| All subjects |  |  |  |  |  |  |  |  |  |  |
| Normal | 26.3/38.2 | 387 (39)/3082 (50) | 23.9/38.2 | 114 (49)/3355 (48) | 28.1/38.2 | 40 (35)/3429 (49) |  |  |  |  |
| Overweight | 27.1/36.2 | 446 (45)/2431 (39) | 24.9/35.2 | 99 (43)/2778 (40) | 28.5/35.2 | 51 (45)/2826 (40) |  |  |  |  |
| Obese | 25.1/32.4 | 158 (16)/683 (11) | 27.5/31.4 | 19 (8)/822 (12) | 27.3/31.3 | 22 (20)/ 819 (12) |  |  |  |  |
|  |  |  |  |  |  |  |  |  |  |  |
| Females |  |  |  |  |  |  |  |  |  |  |
| Normal | 27.1/38.2 | 135 (33)/1504 (49) | 26.2/38.2 | 19 (48)/1620 (47) | 26.7/38.2 | 15 (27)/1624 (48) |  |  | 25.4/38.2 | 35 (34)/1604 (48) |
| Overweight | 26.9/38.2 | 161 (39)/1071 (35) | 25.6/36.2 | 14 (35)/1218 (36) | 28.5/36.3 | 21 (38)/1211 (35) |  |  | 26.9/36.6 | 39 (38)/1193 (35) |
| Obese | 26.9/33.6 | 115 (28)/483 (16) | 31.6/32.2 | 7 (17)/ 591 (17) | 29.0/32.2 | 19 (35)/579 (17) |  |  | 27.1/32.5 | 29 (28)/569 (17) |
|  |  |  |  |  |  |  |  |  |  |  |
| Males |  |  |  |  |  |  |  |  |  |  |
| Normal | 25.5/38.2 | 252 (43)/1578 (50) | 23.3/36.7 | 95 (50)/1735 (49) | 28.3/36.2 | 25 (43)/1805 (49) | 34.5/36.2 | 13 (21)/1817 (50) |  |  |
| Overweight | 27.3/35.2 | 285 (49)/1360 (43) | 24.9/34.5 | 85 (44)/1560 (44) | 28.1/33.8 | 30 (52)/1615 (44) | 29.0/33.8 | 41 (67)/1604 (44) |  |  |
| Obese | 21.3/29.0 | 43 (7)/200 (6) | 16.2/27.5 | 12 (6)/231 (7) | 13.9/27.3 | 3 (5)/240 (7) | 24.9/26.9 | 7 (12)/236 (6) |  |  |

Normal= BMI <25 kg/m^2^, Overweight= BMI 25-30 kg/m^2^, Obese= BMI > 30 kg/m^2^.
