# Supplementary material for: BMI and Lifetime Changes in BMI and Cancer Mortality Risk
Source: PLoS One. 2015 Apr 16;10(4):e0125261. doi: 10.1371/journal.pone.0125261 (PMC4399977; doi:10.1371/journal.pone.0125261)
Supplement: S2 Table — Normal = BMI <25 kg/m2, Overweight = BMI 25–30 kg/m2, Obese = BMI > 30 kg/m2. (DOC) [file pone.0125261.s003.doc]

**S2 Table- Number of subjects and follow-up times (FU) of subjects included in the analyses on the associations between highest and lowest BMI level during study-period and mortality due to any cancer, lung cancer, colorectal cancer, prostate cancer, and breast cancer, in a general population of Vlagtwedde-Vlaardingen during 40 years of follow-up.**

| **Highest BMI level, n (%)** | **Any cancer** | | **Lung cancer** | | **Colorectal cancer** | | **Prostate cancer** | | **Breast cancer** | |
| --- | --- | --- | --- | --- | --- | --- | --- | --- | --- | --- |
|  |  |  |  |  |  |  |  |  |  |  |
|  | **median FU (yr)**  **events/censored** | **N (%) events/censored** | **median FU (yr) events/censored** | **N (%) events/censored** | **median FU (yr) events/censored** | **N (%) events/censored** | **median FU (yr) events/censored** | **N (%) events/censored** | **median FU (yr) events/censored** | **N (%) events/censored** |
| All subjects |  |  |  |  |  |  |  |  |  |  |
| Normal | 17.7/26.2 | 116 (21)/1210 (29) | 18.6/25.1 | 35 (26)/1291 (29) | 21.3/24.2 | 11 (20)/1315 (29) |  |  |  |  |
| Overweight | 17.0/23.2 | 308 (55)/2083 (51) | 15.1/23.2 | 77 (57)/2314 (51) | 18.8/23.2 | 27 (48)/2364 (51) |  |  |  |  |
| Obese | 15.7/23.2 | 137 (24)/809 (20) | 18.4/21.2 | 23 (17)/923 (20) | 19.9/21.2 | 18 (32)/928 (20) |  |  |  |  |
|  |  |  |  |  |  |  |  |  |  |  |
| Females |  |  |  |  |  |  |  |  |  |  |
| Normal | 16.3/26.9 | 33 (15)/646 (32) | 21.0/26.2 | 7 (28)/672 (31) | -/26.2 | 1 (4)/678 (31) |  |  | 16.3/26.2 | 11 (22)/668 (31) |
| Overweight | 18.5/23.2 | 108 (50)/860 (43) | 12.8/23.2 | 11 (44)/957 (44) | 20.9/23.2 | 14 (54)/954 (44) |  |  | 18.8/23.2 | 22 (43)/946 (44) |
| Obese | 16.9/23.2 | 74 (34)/494 (24) | 24.2/23.2 | 7 (28)/561 (26) | 23.8/23.2 | 11 (42)/557 (25) |  |  | 11.6/23.2 | 18 (35)/550 (25) |
|  |  |  |  |  |  |  |  |  |  |  |
| Males |  |  |  |  |  |  |  |  |  |  |
| Normal | 17.7/24.2 | 83 (24)/564 (27) | 18.1/24.1 | 28 (25)/619 (27) | 23.3/23.2 | 10 (33)/637 (26) | 17.8/23.2 | 2 (6)/645 (27) |  |  |
| Overweight | 16.4/23.2 | 200 (58)/1223 (58) | 15.2/23.2 | 66 (60)/1357 (58) | 16.8/23.2 | 13 (43)/1410 (58) | 18.5/23.2 | 24 (75)/1399 (58) |  |  |
| Obese | 13.7/19.4 | 63 (18)/315 (15) | 10.6/19.2 | 16 (15)/362 (15) | 12.1/19.2 | 7 (23)/371 (15) | 15.5/19.2 | 6 (19)/372 (15) |  |  |
|  |  |  |  |  |  |  |  |  |  |  |
| **Lowest BMI level, n (%)** | **median FU (yr)**  **events/censored** | **N (%) events/censored** | **median FU (yr) events/censored** | **N (%) events/censored** | **median FU (yr) events/censored** | **N (%) events/censored** | **median FU (yr) events/censored** | **N (%) events/censored** | **median FU (yr) events/censored** | N (%) events/censored |
|  |  |  |  |  |  |  |  |  |  |  |
| All subjects |  |  |  |  |  |  |  |  |  |  |
| Normal | 22.5/35.2 | 298 (53)/2633 (64) | 21.2/33.2 | 80 (59)/2851 (63) | 22.4/33.2 | 25 (45)/2906 (63) |  |  |  |  |
| Overweight | 22.7/32.2 | 210 (37)/1276 (31) | 24.0/32.2 | 46 (34)/1440 (32) | 24.6/32.2 | 24 (43)/1462 (32) |  |  |  |  |
| Obese | 20.8/31.9 | 53 (9)/193 (5) | 30.2/29.8 | 9 (7)/237 (5) | 19.3/30.0 | 7 (12)/239 (5) |  |  |  |  |
|  |  |  |  |  |  |  |  |  |  |  |
| Females |  |  |  |  |  |  |  |  |  |  |
| Normal | 22.6/33.2 | 97 (45)/1269 (64) | 21.4/33.2 | 13 (52)/1353 (62) | 18.9/33.2 | 8 (31)/1358 (62) |  |  | 21.4/33.2 | 22 (43)/1344 (62) |
| Overweight | 19.4/32.2 | 78 (36)/589 (29) | 24.6/32.2 | 7 (28)/660 (30) | 22.1/32.2 | 11 (42)/656 (30) |  |  | 15.1/32.2 | 19 (37)/648 (30) |
| Obese | 23.6/32.2 | 40 (19)/142 (7) | 30.2/30.5 | 5 (20)/177 (8) | 19.3/31.4 | 7 (27)/175 (8) |  |  | 18.9/31.7 | 10 (20)/172 (8) |
|  |  |  |  |  |  |  |  |  |  |  |
| Males |  |  |  |  |  |  |  |  |  |  |
| Normal | 22.5/35.2 | 201 (58)/1364 (65) | 21.1/34.9 | 67 (61)/1498 (64) | 24.2/33.2 | 17 (57)/1548 (64) | 22.8/33.2 | 15 (47)/1550 (64) |  |  |
| Overweight | 23.8/32.2 | 132 (38)/687 (33) | 23.4/32.2 | 39 (35)/780 (33) | 28.1/32.2 | 13 (43)/806 (33) | 23.7/32.2 | 15 (47)/804 (33) |  |  |
| Obese | 12.7/29.2 | 13 (4)/51 (2) | 20.3/28.1 | 4 (4)/60 (3) | -/28.1 | 0 (0.0)/64 (3) | 23.5/28.1 | 2 (6)/62 (3) |  |  |

Normal= BMI <25 kg/m^2^, Overweight= BMI 25-30 kg/m^2^, Obese= BMI > 30 kg/m^2^.
