# Supplementary material for: BMI and Lifetime Changes in BMI and Cancer Mortality Risk
Source: PLoS One. 2015 Apr 16;10(4):e0125261. doi: 10.1371/journal.pone.0125261 (PMC4399977; doi:10.1371/journal.pone.0125261)
Supplement: S3 Table — Decrease = < .0.02 kg/m2/yr, No change = -0.02–0.02 kg/m2/yr, Moderate increase = 0.02–0.4 kg/m2/yr, High increase = > 0.4 kg/m2/yr. (DOC) [file pone.0125261.s004.doc]

**S3 Table- Number of subjects and follow-up times (FU) of subjects included in the analyses on the associations between long-term annual change in BMI and mortality due to any cancer, lung cancer, colorectal cancer, prostate cancer, and breast cancer, in a general population of Vlagtwedde-Vlaardingen during 40 years of follow-up.**

| **Long-term change in BMI, n (%)** | **Any cancer** | | **Lung cancer** | | **Colorectal cancer** | | **Prostate cancer** | | **Breast cancer** | |
| --- | --- | --- | --- | --- | --- | --- | --- | --- | --- | --- |
|  |  |  |  |  |  |  |  |  |  |  |
|  | **median FU (yr)**  **events/censored** | **N (%) events/censored** | **median FU (yr) events/censored** | **N (%) events/censored** | **median FU (yr) events/censored** | **N (%) events/censored** | **median FU (yr) events/censored** | **N (%) events/censored** | **median FU (yr) events/censored** | **N (%) events/censored** |
| All subjects |  |  |  |  |  |  |  |  |  |  |
| Decrease | 27.4/38.2 | 171 (31)/967 (24) | 27.1/36.2 | 37 (27)/1101 (24) | 31.2/36.1 | 18 (32)/1120 (24) |  |  |  |  |
| No change | 30.7/39.2 | 75 (13)/405 (10) | 26.5/38.3 | 23 (17)/457 (10) | 29.9/38.2 | 4 (7)/476 (10) |  |  |  |  |
| Moderate increase | 30.0/38.2 | 293 (52)/2452 (60) | 29.4/38.2 | 69 (51)/2676 (59) | 32.1/38.2 | 34 (61)/2711 (59) |  |  |  |  |
| High increase | 27.3/35.6 | 22 (4)/278 (7) | 29.1/35.2 | 6 (4)/294 (7) | -/35.2 | 0 (0.0)/300 (7) |  |  |  |  |
|  |  |  |  |  |  |  |  |  |  |  |
| Females |  |  |  |  |  |  |  |  |  |  |
| Decrease | 26.8/38.2 | 75 (35)/535 (27) | 26.9/38.0 | 5 (20)/605 (28) | 31.7/38.1 | 11 (42)/599 (27) |  |  | 22.1/38.2 | 19 (37)/591 (27) |
| No change | 32.0/39.2 | 25 (12)/189 (10) | 34.8/39.2 | 5 (20)/209 (10) | 18.7/39.2 | 2 (8)/212 (10) |  |  | 15.6/39.2 | 4 (8)/210 (10) |
| Moderate increase | 31.0/39.2 | 104 (48)/1127 (56) | 30.6/38.2 | 12 (48)/1219 (56) | 31.7/38.2 | 13 (50)/1218 (56) |  |  | 31.7/38.2 | 25 (49)/1206 (56) |
| High increase | 23.7/36.2 | 11 (5)/149 (7) | 27.5/36.1 | 3 (12)/157 (7) | -/35.2 | 0 (0.0)/160 (7) |  |  | 23.7/36.1 | 3 (6)/157 (7) |
|  |  |  |  |  |  |  |  |  |  |  |
| Males |  |  |  |  |  |  |  |  |  |  |
| Decrease | 28.7/36.2 | 96 (28)/432 (21) | 27.7/35.2 | 32 (29)/496 (21) | 30.3/35.2 | 7 (23)/521 (21) | 30.8/35.2 | 10 (31)/518 (21) |  |  |
| No change | 28.8/38.2 | 50 (15)/216 (10) | 25.8/38.2 | 18 (16)/248 (11) | 34.3/38.2 | 2 (7)/264 (11) | 28.5/38.2 | 6 (19)/260 (11) |  |  |
| Moderate increase | 29.7/38.2 | 189 (55)/1325 (63) | 28.4/38.2 | 57 (52)/1457 (62) | 33.8/38.2 | 21 (70)/1493 (62) | 34.9/38.2 | 15 (47)/1499 (62) |  |  |
| High increase | 32.1/35.2 | 11 (3)/129 (6) | 30.7/35.2 | 3 (3)/137 (6) | -/35.2 | 0 (0)/140 (6) | -/35.2 | 1 (3)/139 (6) |  |  |
|  |  |  |  |  |  |  |  |  |  |  |

Decrease= < -0.02 kg/m^2^/yr, No change= -0.02-0.02 kg/m^2^/yr, Moderate increase= 0.02-0.4 kg/m^2^/yr, High increase= > 0.4 kg/m^2^/yr.
