# Supplementary material for: BMI and Lifetime Changes in BMI and Cancer Mortality Risk
Source: PLoS One. 2015 Apr 16;10(4):e0125261. doi: 10.1371/journal.pone.0125261 (PMC4399977; doi:10.1371/journal.pone.0125261)
Supplement: S4 Table — Highest short-term annual increase in BMI: No increase = < 0.10 kg/m2/yr, Moderate increase = 0.10–0.50 kg/m2/yr, High increase = > 0.50 kg/m2/yr. Highest short-term annual decrease in BMI: No decrease = > -0.10 kg/m2/yr, Moderate decrease = -0.10- -0.50 kg/m2/yr, High decrease = < -0.50 kg/m2/yr. (DOC) [file pone.0125261.s005.doc]

**S4 Table- Number of subjects and follow-up times (FU) of subjects included in the analyses on the associations between short-term annual changes in BMI and mortality due to any cancer, lung cancer, colorectal cancer, prostate cancer, and breast cancer, in a general population of Vlagtwedde-Vlaardingen during 40 years of follow-up.**

| **Highest short-term annual increase in BMI, n (%)** | **Any cancer** | | **Lung cancer** | | **Colorectal cancer** | | **Prostate cancer** | | **Breast cancer** | |
| --- | --- | --- | --- | --- | --- | --- | --- | --- | --- | --- |
|  |  |  |  |  |  |  |  |  |  |  |
|  | **median FU (yr)**  **events/censored** | **N (%) events/censored** | **median FU (yr) events/censored** | **N (%) events/censored** | **median FU (yr) events/censored** | **N (%) events/censored** | **median FU (yr) events/censored** | **N (%) events/censored** | **median FU (yr) events/censored** | **N (%) events/censored** |
| All subjects |  |  |  |  |  |  |  |  |  |  |
| No increase | 18.6/29.2 | 40 (8)/156 (5) | 19.9/28.2 | 8 (7)/188 (5) | 24.5/27.6 | 4 (9)/192 (5) |  |  |  |  |
| Moderate increase | 20.7/29.4 | 237 (50)/1605 (47) | 19.8/29.2 | 68 (59)/1774 (47) | 21.3/29.2 | 23 (51)/1819 (48) |  |  |  |  |
| High increase | 22.2/30.2 | 198 (42)/1628 (48) | 20.2/30.2 | 40 (35)/1786 (48) | 19.7/30.1 | 18 (40)/1808 (47) |  |  |  |  |
|  |  |  |  |  |  |  |  |  |  |  |
| Females |  |  |  |  |  |  |  |  |  |  |
| No increase | 18.4/32.2 | 18 (10)/87 (5) | 21.2/29.2 | 2 (9)/103 (6) | 20.8/29.2 | 2 (10)/103 (6) |  |  | 18.6/29.5 | 6 (15)/99 (6) |
| Moderate increase | 24.6/29.7 | 85 (48)/726 (44) | 30.4/29.2 | 8 (36)/803 (45) | 19.8/29.2 | 11 (55)/800 (45) |  |  | 25.5/29.2 | 16 (40)/795 (45) |
| High increase | 22.4/30.2 | 76 (42)/822 (50) | 22.7/29.2 | 12 (55)/886 (49) | 23.7/29.2 | 7 (35)/891 (50) |  |  | 21.9/29.2 | 18 (45)/880 (50) |
|  |  |  |  |  |  |  |  |  |  |  |
| Males |  |  |  |  |  |  |  |  |  |  |
| No increase | 18.9/27.2 | 22 (7)/69 (4) | 19.9/26.2 | 6 (6)/85 (4) | 34.1/24.2 | 2 (8)/89 (4) | 21.6/25.8 | 4 (14)/87 (4) |  |  |
| Moderate increase | 19.5/29.2 | 152 (51)/879 (50) | 19.3/29.2 | 60 (64)/971 (50) | 22.0/29.2 | 12 (48)/1019 (50) | 18.3/29.2 | 14 (48)/1017 (50) |  |  |
| High increase | 21.4/32.2 | 122 (41)/806 (46) | 19.7/30.2 | 28 (30)/900 (46) | 18.3/30.2 | 11 (44)/917 (45) | 25.0/30.2 | 11 (38)/917 (45) |  |  |
|  |  |  |  |  |  |  |  |  |  |  |
| **Highest short-term annual decrease in BMI, n (%)** | **median FU (yr)**  **events/censored** | **N (%) events/censored** | **median FU (yr) events/censored** | **N (%) events/censored** | **median FU (yr) events/censored** | **N (%) events/censored** | **median FU (yr) events/censored** | **N (%) events/censored** | **median FU (yr) events/censored** | **N (%) events/censored** |
|  |  |  |  |  |  |  |  |  |  |  |
| All subjects |  |  |  |  |  |  |  |  |  |  |
| No decrease | 21.3/32.2 | 76 (16)/619 (18) | 20.3/30.2 | 20 (17)/675 (18) | 11.6/30.2 | 4 (9)/691 (18) |  |  |  |  |
| Moderate increase | 23.2/32.2 | 256 (54)/1722 (51) | 22.5/30.2 | 64 (55)/1914 (51) | 25.5/30.2 | 24 (53)/1954 (51) |  |  |  |  |
| High decrease | 21.5/32.2 | 143 (30)/1048 (31) | 22.3/30.3 | 32 (28)/1159 (31) | 25.2/30.2 | 17 (38)/1174 (31) |  |  |  |  |
|  |  |  |  |  |  |  |  |  |  |  |
| Females |  |  |  |  |  |  |  |  |  |  |
| No decrease | 22.9/32.2 | 29 (16)/253 (16) | 26.6/32.2 | 6 (27)/276 (15) | 15.1/32.2 | 3 (15)/279 (16) |  |  | 22.4/32.2 | 9 (23)/273 (15) |
| Moderate increase | 26.3/32.2 | 85 (48)/781 (48) | 27.4/32.2 | 7 (32)/859 (48) | 23.8/32.2 | 9 (45)/857 (48) |  |  | 27.4/32.2 | 16 (40)/850 (48) |
| High decrease | 19.5/32.2 | 65 (36)/601 (37) | 23.9/32.2 | 9 (41)/657 (37) | 22.9/32.2 | 8 (40)/658 (37) |  |  | 18.3/32.2 | 15 (37)/651 (37) |
|  |  |  |  |  |  |  |  |  |  |  |
| Males |  |  |  |  |  |  |  |  |  |  |
| No decrease | 20.9/30.2 | 47 (16)/366 (21) | 14.7/29.4 | 14 (15)/399 (20) | -/29.2 | 1 (4)/412 (20) | 25.0/29.2 | 4 (14)/409 (20) |  |  |
| Moderate increase | 22.5/30.2 | 171 (58)/941 (54) | 22.5/29.2 | 57 (61)/1055 (54) | 25.5/29.2 | 15 (60)/1097 (54) | 25.3/29.2 | 19 (65)/1093 (54) |  |  |
| High decrease | 23.7/30.2 | 78 (26)/447 (25) | 22.2/29.7 | 23 (24)/502 (26) | 27.3/29.2 | 9 (36)/516 (26) | 33.6/29.2 | 6 (21)/519 (26) |  |  |

Highest short-term annual increase in BMI: No increase= < 0.10 kg/m^2^/yr, Moderate increase= 0.10-0.50 kg/m^2^/yr, High increase= > 0.50 kg/m^2^/yr. Highest short-term annual decrease in BMI: No decrease= > -0.10 kg/m^2^/yr, Moderate decrease= -0.10- -0.50 kg/m^2^/yr, High decrease= < -0.50 kg/m^2^/yr.
