# Supplementary material for: BMI and Lifetime Changes in BMI and Cancer Mortality Risk
Source: PLoS One. 2015 Apr 16;10(4):e0125261. doi: 10.1371/journal.pone.0125261 (PMC4399977; doi:10.1371/journal.pone.0125261)
Supplement: S5 Table — Long-term annual change in BMI: The difference between BMI at last survey and baseline divided by the time interval (year of the last survey minus year of the baseline). Short-term annual changes in BMI: Highest increase = Highest annual increase in BMI between two successive surveys, Highest decrease = Highest annual decrease between two successive surveys. Data on BMI levels and changes in BMI are shown as mean (sd). (DOC) [file pone.0125261.s006.doc]

**S5 Table- Characteristics at baseline, for subjects who died due to lung cancer, colorectal cancer, prostate cancer, and breast cancer in a general population of Vlagtwedde-Vlaardingen during 40 years of follow-up.**

| **Characteristics for all subjects** | **Any cancer**  **(n=1194)** | **Lung cancer**  **(n=275)** | **Colorectal cancer**  **(n=134)** | **Prostate cancer**  **(n=83)** | **Breast cancer**  **(n=117)** |
| --- | --- | --- | --- | --- | --- |
|  |  |  |  |  |  |
| All subjects (%) | 14.1 | 3.2 | 1.6 | 1.0 | 1.4 |
|  |  |  |  |  |  |
| Men (%) | 58.3 | 83.3 | 49.3 | 100 | 0.0 |
|  |  |  |  |  |  |
| Age in years, mean (SD) | 45.9 (11.1) | 40.8 (8.4) | 44.0 (8.6) | 47.7 (10.2) | 39.7 (11.2) |
|  |  |  |  |  |  |
| Smoking at first visit, n (%) |  |  |  |  |  |
| Never smokers | 392 (33.2) | 6 (4.3) | 20 (36.4) | 14 (16.9) | 34 (66.7) |
| Ever smokers | 787 (66.8) | 132 (95.7) | 35 (63.6) | 69 (83.1) | 17 (33.3) |
|  |  |  |  |  |  |
| BMI levels, kg/m^2^ |  |  |  |  |  |
| BMI at baseline | 26.3 (3.8) | 25.4 (3.2) | 27.0 (4.2) | 26.4 (2.8) | 27.3 (4.3) |
| Highest BMI during follow-up | 27.9 (4.0)  25.2 (3.6) | 27.2 (3.4)  24.6 (3.3) | 28.9 (4.9)  25.9 (4.0) | 27.7 (3.0)  26.0 (2.5) | 29.0 (5.1)  26.3 (4.6) |
| Lowest BMI during follow-up | 25.2 (3.6)  25.2 (3.6) | 24.6 (3.3) | 25.9 (4.0) | 26.0 (2.5) | 26.3 (4.6) |
|  |  |  |  |  |  |
| Long term annual change in BMI, kg/m^2^/yr | 0.1 (0.2) | 0.1 (0.2) | 0.0 (0.2) | 0.0 (0.2) | 0.1 (0.2) |
|  |  |  |  |  |  |
| Short-term annual change in BMI, kg/m^2^/yr |  |  |  |  |  |
| Highest increase | 0.4 (0.4)  -0.4 (0.4) | 0.4 (0.3) | 0.4 (0.5) | 0.4 (0.3) | 0.4 (0.4) |
| Highest decrease | -0.3 (0.4) | -0.3 (0.4) | -0.3 (0.4) | -0.3 (0.3) | -0.3 (0.5) |
|  |  |  |  |  |  |

Long-term annual change in BMI: The difference between BMI at last survey and baseline divided by the time interval (year of the last survey minus year of the baseline). Short-term annual changes in BMI: Highest increase=Highest annual increase in BMI between two successive surveys, Highest decrease=Highest annual decrease between two successive surveys. Data on BMI levels and changes in BMI are shown as mean (sd).
