# Supplementary material for: BMI and Lifetime Changes in BMI and Cancer Mortality Risk
Source: PLoS One. 2015 Apr 16;10(4):e0125261. doi: 10.1371/journal.pone.0125261 (PMC4399977; doi:10.1371/journal.pone.0125261)
Supplement: S6 Table — Stratification according to sex and interactions are shown. Normal = BMI <25 kg/m2, overweight = BMI 25–30 kg/m2, obese = BMI > 30 kg/m2. Statistically significant results are shown in bold. (DOC) [file pone.0125261.s007.doc]

**S6 Table -** **Hazard ratio (with 95% confidence interval) of BMI at baseline for mortality from any cancer, lung cancer, colorectal cancer, among 3718 males and 3469 females in Cox regression with adjustment for age, smoking habits, and place of residence. Stratification according to sex and interactions are shown.**

| **BMI level at baseline** | **Any cancer**  **HR (95% CI)** | **Lung cancer**    **HR (95% CI)** | **Colorectal cancer**  **HR (95% CI)** |
| --- | --- | --- | --- |
| Females |  |  |  |
| Normal | 1 | 1 | 1 |
| Overweight | 0.96 (0.75-1.21) | 0.81 (0.39-1.69) | 0.86 (0.43-1.70) |
| Obese | **1.40 (1.07-1.84)** | 0.98 (0.38-2.50) | 1.67 (0.81-3.45) |
|  |  |  |  |
| Males |  |  |  |
| Normal | 1 | 1 | 1 |
| Overweight | 0.88 (0.74-1.05) | **0.69 (0.51-0.92)** | 0.85 (0.50-1.46) |
| Obese | 1.06 (0.76-1.47) | 0.73 (0.40-1.35) | 0.70 (0.20-2.23) |
|  |  |  |  |
| Interaction |  |  |  |
| Normal | 1 | 1 | 1 |
| Overweight | 1.01 (0.76-1.34) | 1.02 (0.48-2.16) | 1.00 (0.43-2.36) |
| Obese | 0.81 (0.54-1.22) | 0.84 (0.29-2.45) | 0.45 (0.11-1.78) |
|  |  |  |  |
|  |  |  |  |

Normal= BMI <25 kg/m^2^, overweight= BMI 25-30 kg/m^2^, obese= BMI > 30 kg/m^2^. Statistically significant results are shown in bold.
