# Supplementary material for: BMI and Lifetime Changes in BMI and Cancer Mortality Risk
Source: PLoS One. 2015 Apr 16;10(4):e0125261. doi: 10.1371/journal.pone.0125261 (PMC4399977; doi:10.1371/journal.pone.0125261)
Supplement: S7 Table — Stratification according to sex and interactions are shown. BMI levels: Normal = BMI <25 kg/m2, overweight = BMI 25–30 kg/m2, obese = BMI > 30 kg/m2. Statistically significant results are shown in bold. NA: Not Available, no mortality in this category. (DOC) [file pone.0125261.s008.doc]

**S7 Table- Hazard ratio (with 95% confidence interval) of highest and lowest BMI during the study-period for mortality from any cancer, lung cancer , colorectal cancer among 2448 males and 2215 females in Cox regression with adjustment for age, smoking habits, and place of residence.** **Stratification according to sex and interactions are shown.**

| **Highest BMI level** | **Any cancer**  **HR (95% CI)** | **Lung cancer**  **HR (95% CI)** | **Colorectal cancer**  **HR (95% CI)** |
| --- | --- | --- | --- |
|  |  |  |  |
| Females |  |  |  |
| Normal | 1 | 1 | 1 |
| Overweight | **1.59 (1.06-2.38)** | 0.89 (0.33-2.40) | 4.92 (0.64-38.14) |
| Obese | **1.87 (1.21-2.89)** | 1.06 (0.35-3.27) | 7.21 (0.90-57.61) |
|  |  |  |  |
| Males |  |  |  |
| Normal | 1 | 1 | 1 |
| Overweight | 0.80 (0.62-1.03) | 0.76 (0.48-1.18) | **0.40 (0.18-0.93)** |
| Obese | 1.07 (0.77-1.50) | 0.75 (0.40-1.40) | 0.91 (0.34-2.45) |
|  |  |  |  |
| Interaction |  |  |  |
| Normal | 1 | 1 | 1 |
| Overweight | **0.58 (0.36-0.93)** | 1.01 (0.35-2.88) | **0.09 (0.01-0.77)** |
| Obese | 0.67 (0.40-1.14) | 0.83 (0.25-2.84) | 0.16 (0.02-1.59) |

| **Lowest BMI level** | **Any cancer**  **HR (95% CI)** | **Lung cancer**  **HR (95% CI)** | **Colorectal cancer**  **HR (95% CI)** |
| --- | --- | --- | --- |
|  |  |  |  |
| Females |  |  |  |
| Normal | 1 | 1 | 1 |
| Overweight | 1.07 (0.79-1.46) | 0.96 (0.37-2.51) | 1.69 (0.66-4.29) |
| Obese | **2.16 (1.47-3.18)** | **3.22 (1.06-9.76)** | **4.32 (1.53-12.20)** |
|  |  |  |  |
| Males |  |  |  |
| Normal | 1 | 1 | 1 |
| Overweight | 0.91 (0.73-1.14) | 0.78 (0.52-1.17) | 0.96 (0.46-1.99) |
| Obese | 1.50 (0.86-2.64) | 1.31 (0.48-3.61) | NA |
|  |  |  |  |
| Interaction |  |  |  |
| Normal | 1 | 1 | 1 |
| Overweight | 0.91 (0.63-1.33) | 0.98 (0.36-2.67) | 0.68 (0.21-2.19) |
| Obese | 0.74 (0.38-1.45) | 0.54 (0.13-2.31) | NA |
|  |  |  |  |

BMI levels: Normal= BMI <25 kg/m^2^, overweight= BMI 25-30 kg/m^2^, obese= BMI > 30 kg/m^2^. Statistically significant results are shown in bold. NA: Not Available, no mortality in this category.
