# Supplementary material for: BMI and Lifetime Changes in BMI and Cancer Mortality Risk
Source: PLoS One. 2015 Apr 16;10(4):e0125261. doi: 10.1371/journal.pone.0125261 (PMC4399977; doi:10.1371/journal.pone.0125261)
Supplement: S8 Table — Prostate cancer among 2448 males and breast cancer among 2215 females in Cox regression with adjustment for age, smoking habits, and place of residence. Long-term annual change in BMI: Decrease = < -0.02 kg/m2/yr, no change = -0.02–0.02 kg/m2/yr, moderate increase = 0.02–0.4 kg/m2/yr, high increase = > 0.4 kg/m2/yr. NA: Not Available, no mortality in this category. (DOC) [file pone.0125261.s009.doc]

**S8 Table- Hazard ratio (with 95% confidence interval) of long-term annual change in BMI categories for mortality from all cancer, lung cancer, colorectal cancer among all 4663 subjects. Prostate cancer among 2448 males and breast cancer among 2215 females in Cox regression with adjustment for age, smoking habits, and place of residence.**

| **Annual change in BMI over the entire study period** | **Any cancer**  **HR (95% CI)** | **Lung cancer**  **HR (95% CI)** | **Colorectal cancer**  **HR (95% CI)** | **Prostate cancer**  **HR (95% CI)** | **Breast cancer**  **HR (95% CI)** |
| --- | --- | --- | --- | --- | --- |
|  |  |  |  |  |  |
| All subjects |  |  |  |  |  |
| Decrease | 0.99 (0.75-1.30) | 0.75 (0.44-1.27) | 1.81 (0.61-5.38) | 0.78 (0.28-2.17) | 1.65 (0.56-4.90) |
| No change | 1 | 1 | 1 | 1 | 1 |
| Moderate increase | 0.95 (0.74-1.23) | 0.73 (0.45-1.17) | 2.26 (0.80-6.39) | 0.71 (0.27-1.84) | 1.34 (0.46-3.87) |
| High increase | 1.24 (0.76-2.00) | 1.10 (0.44-2.75) | NA | 1.54 (0.18-13.25) | 1.86 (0.41-8.46) |
|  |  |  |  |  |  |
|  |  |  |  |  |  |

Long-term annual change in BMI: Decrease= < -0.02 kg/m^2^/yr, no change= -0.02-0.02 kg/m^2^/yr, moderate increase= 0.02-0.4 kg/m^2^/yr, high increase= > 0.4 kg/m^2^/yr. NA: Not Available, no mortality in this category.
