# Supplementary material for: BMI and Lifetime Changes in BMI and Cancer Mortality Risk
Source: PLoS One. 2015 Apr 16;10(4):e0125261. doi: 10.1371/journal.pone.0125261 (PMC4399977; doi:10.1371/journal.pone.0125261)
Supplement: S9 Table — Stratification according to sex and interactions are shown. Long-term annual change in BMI: Decrease = < -0.02 kg/m2/yr, no change = -0.02–0.02 kg/m2/yr, moderate increase = 0.02–0.4 kg/m2/yr, high increase = > 0.4 kg/m2/yr. NA: Not Available, no mortality in this category. (DOC) [file pone.0125261.s010.doc]

**S9 Table- Hazard ratio (with 95% confidence interval) of long-term annual change in BMI over the entire study period categories for mortality from all cancer, lung cancer, colorectal cancer among 2448 males and 2215 females in Cox regression with adjustment for age, smoking habits, and place of residence. Stratification according to sex and interactions are shown.**

| **Annual change in BMI over the entire study period** | **Any cancer**  **HR (95% CI)** | **Lung cancer**  **HR (95% CI)** | **Colorectal cancer**  **HR (95% CI)** |
| --- | --- | --- | --- |
| Females |  |  |  |
| Decrease | 1.01 (0.64-1.60) | 0.34 (0.10-1.17) | 1.65 (0.36-7.56) |
| No change | 1 | 1 | 1 |
| Moderate increase | 0.96 (0.62-1.49) | 0.46 (0.16-1.32) | 1.87 (0.42-8.32) |
| High increase | 1.30 (0.63-2.66) | 1.14 (0.26-4.97) | NA |
|  |  |  |  |
| Males |  |  |  |
| Decrease | 0.98 (0.69-1.380 | 0.90 (0.50-1.60) | 1.75 (0.36-8.48) |
| No change | 1 | 1 | 1 |
| Moderate increase | 0.95 (0.70-1.31) | 0.80 (0.47-1.37) | 2.64 (0.62-11.34) |
| High increase | 1.19 (0.61-2.30) | 0.87 (0.25-2.99) | NA |
|  |  |  |  |
| Interaction |  |  |  |
| Decrease | 0.97 (0.55-1.71) | 2.85 (0.73-11.20) | 0.98 (0.11-8.65) |
| No change | 1 | 1 | 1 |
| Moderate increase | 0.97 (0.56-1.65) | 1.59 (0.49-5.11) | 1.67 (0.21-13.35) |
| High increase | 0.85 (0.32-2.22) | 0.63 (0.10-4.17) | NA |
|  |  |  |  |

Long-term annual change in BMI: Decrease= < -0.02 kg/m^2^/yr, no change= -0.02-0.02 kg/m^2^/yr, moderate increase= 0.02-0.4 kg/m^2^/yr, high increase= > 0.4 kg/m^2^/yr. NA: Not Available, no mortality in this category.
