# Supplementary material for: BMI and Lifetime Changes in BMI and Cancer Mortality Risk
Source: PLoS One. 2015 Apr 16;10(4):e0125261. doi: 10.1371/journal.pone.0125261 (PMC4399977; doi:10.1371/journal.pone.0125261)
Supplement: S10 Table — Stratification according to sexes and interactions are shown. Normal = BMI <25 kg/m2, Overweight = BMI 25–30 kg/m2, Obese = BMI > 30 kg/m2. (DOC) [file pone.0125261.s011.doc]

**S10 Table - Hazard ratio (with 95% confidence interval) of short-term annual changes in BMI (highest increase and highest decrease in BMI between two subsequent observations) for mortality from all cancer, lung cancer, colorectal cancer among 2050 males 1814 females in Cox regression with adjustment for age, smoking habits, and place of residence. Stratification according to sexes and interactions are shown.**

| **Highest increase in BMI** | **Any cancer**  **HR (95% CI)** | **Lung cancer**  **HR (95% CI)** | **Colorectal cancer**  **HR (95% CI)** |
| --- | --- | --- | --- |
| Females |  |  |  |
| No increase | 1 | 1 | 1 |
| Moderate increase | 0.69 (0.41-1.15) | 0.62 (0.13-2.95) | 0.89 (0.20-4.10) |
| High increase | 0.67 (0.40-1.13) | 0.90 (0.20-4.09) | 0.83 (0.17-4.13) |
|  |  |  |  |
| Males |  |  |  |
| No increase | 1 | 1 | 1 |
| Moderate increase | 0.64 (0.41-1.01) | 0.91 (0.39-2.10) | 0.59 (0.13-2.66) |
| High increase | 0.66 (0.42-1.04) | 0.53 (0.22-1.29) | 0.72 (0.16-3.28) |
|  |  |  |  |
| Interaction |  |  |  |
| No increase | 1 | 1 | 1 |
| Moderate increase | 0.94 (0.45-1.85) | 1.51 (0.26-8.79) | 0.70 (0.08-5.88) |
| High increase | 0.95 (0.48-1.90) | 0.58 (0.10-3.28) | 1.07 (0.12-9.55) |
|  |  |  |  |
| **Highest decrease in BMI** | Any cancer  HR (95% CI) | Lung cancer  HR (95% CI) | Colorectal cancer  HR (95% CI) |
| Females |  |  |  |
| No decrease | 1 | 1 | 1 |
| Moderate decrease | 0.69 (0.45-1.06) | **0.32 (0.11-0.94)** | 0.53 (0.14-1.99) |
| High decrease | 0.70 (0.45-1.09) | 0.54 (0.19-1.53) | 0.66 (0.17-2.57) |
|  |  |  |  |
| Males |  |  |  |
| No decrease | 1 | 1 | 1 |
| Moderate decrease | 0.78 (0.56-1.08) | 0.86 (0.47-1.56) | 3.07 (0.40-23.51) |
| High decrease | 0.75 (0.52-1.09) | 0.73 (0.37-1.43) | 3.74 (0.47-29.87) |
|  |  |  |  |
| Interactions |  |  |  |
| No decrease | 1 | 1 | 1 |
| Moderate decrease | 1.17 (0.69-1.98) | 3.13 (0.91-10.80) | 4.34 (0.39-48.52) |
| High decrease | 1.14 (0.65-2.01) | 1.64 (0.48-5.60) | 4.96 (0.43-58.01) |
|  |  |  |  |

Highest increase in BMI: No increase= < 0.10 kg/m^2^/yr, moderate increase= 0.10-0.50 kg/m^2^/yr, high increase= > 0.50 kg/m^2^/yr. Highest decrease in BMI: No decrease= > -0.10 kg/m^2^/yr, moderate decrease= -0.10- -0.50 kg/m^2^/yr, high decrease= < -0.50 kg/m^2^/yr. Statistically significant results are shown in bold.
