# Supplementary material for: BMI and Lifetime Changes in BMI and Cancer Mortality Risk
Source: PLoS One. 2015 Apr 16;10(4):e0125261. doi: 10.1371/journal.pone.0125261 (PMC4399977; doi:10.1371/journal.pone.0125261)
Supplement: S11 Table — Highest increase in BMI: No increase = < 0.10 kg/m2/yr, moderate increase = 0.10–0.50 kg/m2/yr, high increase = > 0.50 kg/m2/yr. Highest decrease in BMI: No decrease = > -0.10 kg/m2/yr, moderate decrease = -0.10- -0.50 kg/m2/yr, high decrease = < -0.50 kg/m2/yr. Statistically significant results are shown in bold. (DOC) [file pone.0125261.s012.doc]

**S11 Table-** **Competing risk analysis on the association between the lowest BMI during follow-up and cancer, and cardiovascular mortality in males and females.**

| **Lowest BMI level** | **Cancer mortality**  **HR (95% CI)** | **CVD mortality**  **HR (95% CI)** | **Difference**  **HR (95% CI)** |
| --- | --- | --- | --- |
| Females |  |  |  |
| Normal | 1 | 1 | 1 |
| Overweight | 0.97 (0.71-1.31) | **1.35 (1.03-1.78)** | 1.40 (0.94-2.09) |
| Obese | **1.92 (1.32-2.80)** | 1.43 (0.95-2.16) | 0.74 (0.43-1.29) |
|  |  |  |  |
| Males |  |  |  |
| Normal | 1 | 1 | 1 |
| Overweight | 0.90 (0.73-1.13) | 1.00 (0.80-1.22) | 1.09 (0.81-1.48) |
| Obese | 1.50 (0.86-2.64) | **1.85 (1.12-3.03)** | 1.22 (0.58-2.60) |
|  |  |  |  |

Normal= BMI <25 kg/m^2^, Overweight= BMI 25-30 kg/m^2^, Obese= BMI > 30 kg/m^2^.
